# Association of Sleep Characteristics With Suspected Metabolic Dysfunction-Associated Fatty Liver Disease (MAFLD) Risk Among Adults Attending a Community Health Center

**DOI:** 10.7759/cureus.111701

**Published:** 2026-06-29

**Authors:** Tushar Prabhakar, Gahan S Jois, Satya V Singh, Bronica Saini, Adarsh Mohan

**Affiliations:** 1 Community Medicine, Amrita School of Medicine, Faridabad, IND; 2 Obstetrics and Gynecology, Employees' State Insurance Corporation (ESIC) Medical College and Hospital, Faridabad, IND

**Keywords:** fatty liver disease, mafld, pittsburgh sleep quality index, risk assessment, sleep quality

## Abstract

Introduction: Metabolic dysfunction-associated fatty liver disease (MAFLD) is a growing public health concern worldwide. Emerging evidence suggests that sleep disturbances may contribute to metabolic dysfunction and hepatic steatosis; however, data from rural Indian populations remain limited. This study assessed the association between sleep quality, sleep duration, and MAFLD risk among adults attending a rural primary healthcare facility in North India.

Methods: A community-based analytical cross-sectional study was conducted among 550 adults attending the Rural Health Training Center of a tertiary care teaching institution in Haryana, India. Sleep quality was assessed using the Pittsburgh Sleep Quality Index (PSQI), and participants were classified as having good (PSQI ≤ 5) or poor (PSQI > 5) sleep quality. Sleep duration was categorized as <6 hours, 6-8 hours, or >8 hours per night. MAFLD risk was estimated using a validated non-laboratory risk assessment score and categorized as low (<8 points) or high (≥8 points). Multivariable logistic regression was performed to evaluate the association of sleep-related factors with high MAFLD risk after adjustment for sex, smoking status, alcohol consumption, and dietary pattern.

Results: The mean age of participants was 42.6 ± 13.5 years, and 56.4% were female. Poor sleep quality was observed in 210 (38.2%) participants, while 174 (31.6%) were classified as having high MAFLD risk. High MAFLD risk was significantly more common among participants with poor sleep quality than among those with good sleep quality (47.1% vs. 22.1%; p < 0.001). Participants sleeping <6 hours per night had the highest prevalence of high MAFLD risk (44.6%) compared with those sleeping 6-8 hours (28.5%) or >8 hours (26.4%) (p = 0.002). After adjustment for sex, smoking status, alcohol consumption, and dietary pattern, poor sleep quality (adjusted odds ratio (AOR): 2.68; 95% CI: 1.82-3.95) and short sleep duration (AOR: 1.71; 95% CI: 1.10-2.64) remained independently associated with high MAFLD risk. The PSQI score demonstrated acceptable discriminatory ability for identifying high MAFLD risk (AUC = 0.71; 95% CI: 0.65-0.77).

Conclusions: Poor sleep quality and short sleep duration were independently associated with high MAFLD risk among adults in a rural North Indian primary care setting. These associations remained significant after adjustment for selected demographic and lifestyle factors. Incorporating sleep assessment into routine metabolic risk screening may provide a simple, low-cost strategy for identifying individuals at increased risk of MAFLD.

## Introduction

Metabolic dysfunction-associated fatty liver disease (MAFLD) is currently among the most prevalent chronic liver disorders globally and has become a significant public health concern [1]. The condition is strongly associated with obesity, insulin resistance, type 2 diabetes mellitus, dyslipidemia, and other metabolic abnormalities. MAFLD represents a broad disease spectrum that ranges from isolated hepatic fat accumulation to steatohepatitis, progressive fibrosis, cirrhosis, and, in advanced cases, hepatocellular carcinoma [2,3]. Recent estimates suggest that nearly one-third of the global population may be affected by steatotic liver disease, with prevalence increasing steadily across both developed and developing countries [4]. In India, the burden of MAFLD is rising rapidly, owing to ongoing epidemiological transition, increasing sedentary lifestyles, dietary changes, and growing prevalence of metabolic disorders.

Recent evidence suggests that MAFLD is not confined to obese individuals and may also occur in metabolically vulnerable individuals with normal body mass index. Community-based studies from India have reported a substantial burden of fatty liver disease, including among lean individuals, highlighting the need for improved risk stratification and early identification strategies [5]. Given the asymptomatic nature of early disease, many individuals remain undiagnosed until advanced stages, underscoring the importance of identifying modifiable risk factors that can be targeted through preventive interventions [6].

Sleep is increasingly recognized as an important determinant of metabolic health. Adequate sleep plays a critical role in maintaining glucose homeostasis, hormonal regulation, appetite control, energy balance, and circadian rhythm synchronization. Disturbances in sleep quality or duration have been associated with obesity, diabetes mellitus, hypertension, cardiovascular disease, and metabolic syndrome. Consequently, sleep-related factors have attracted growing attention as potential contributors to the development of MAFLD [7,8].

Several pathophysiological pathways have been proposed to explain the association between sleep disturbances and the development of hepatic steatosis. Sleep deprivation has been shown to promote insulin resistance, impair glucose metabolism, increase sympathetic nervous system activity, and alter the secretion of appetite-regulating hormones such as leptin and ghrelin [9]. Inadequate sleep is also associated with increased systemic inflammation, characterized by elevated concentrations of inflammatory mediators, including interleukin-6 and C-reactive protein. Furthermore, disruption of circadian rhythms may alter hepatic lipid metabolism through dysregulation of clock genes involved in lipid synthesis, oxidation, and storage. These pathways collectively contribute to metabolic dysfunction and may increase susceptibility to MAFLD [9,10].

Previous epidemiological studies have reported significant associations between poor sleep quality, inadequate sleep duration, and an increased risk of fatty liver disease [11-13]. Longitudinal studies have reported that inadequate sleep may independently predict incident steatotic liver disease after adjustment for conventional metabolic risk factors. However, findings across studies remain heterogeneous, and much of the available evidence originates from urban populations or high-income countries. Data from rural populations in low- and middle-income settings remain limited despite the growing burden of metabolic disease in these communities.

From a public health perspective, identifying simple and scalable approaches for MAFLD risk assessment is particularly important in primary healthcare settings where access to imaging and laboratory investigations may be limited. Assessment of sleep quality using the Pittsburgh Sleep Quality Index (PSQI) and estimation of MAFLD risk using validated non-laboratory risk scores offer practical and cost-effective tools for community-based screening [14,15]. If sleep-related parameters are shown to be associated with MAFLD risk, they may provide an opportunity for early identification of high-risk individuals and implementation of lifestyle interventions before the onset of clinically significant liver disease.

The present study aimed to assess the association between sleep duration and quality and risk of MAFLD among adults attending a rural primary healthcare facility in North India. Additionally, the study sought to identify independent predictors of high MAFLD risk and evaluate the potential utility of sleep-related measures as screening indicators in resource-constrained settings.

A preliminary version of this work was published as a conference abstract in the EASL Congress 2026 abstract supplement (Abstract ID: SAT-011) and was subsequently presented as an oral abstract at the 35th Annual Meeting of the Asian Pacific Association for the Study of the Liver (APASL 2026), Istanbul, Türkiye.

## Materials and methods

Study setting

A community-based analytical cross-sectional study was conducted among adults attending the outpatient department of the Rural Health Training Center (RHTC), the field practice area of the Department of Community Medicine of a medical college in Faridabad, Haryana, India. The study was conducted over a period of six months, including five months of participant recruitment and data collection.

Study population and eligibility criteria

Adults aged 18 years and above attending the RHTC outpatient department during the study period were eligible for inclusion. Participants were excluded if they had a known history of chronic liver disease of non-metabolic etiology, current pregnancy, severe psychiatric illness impairing reliable questionnaire administration, acute medical illness requiring urgent referral, or inability to provide informed consent.

Sample size calculation

The sample size was calculated using the formula for comparison of two proportions [16]. Assuming the prevalence of high MAFLD risk among poor sleepers to be 18% and among good sleepers to be 10%, with a confidence level of 95% and statistical power of 80%, the minimum required sample size was estimated to be 490 participants [11]. To compensate for an expected non-response rate of 10%, the final sample size was increased to 550 participants.

Sampling and data collection

A consecutive sampling technique was employed. All adults aged ≥18 years attending the outpatient department of the Rural Health Training Centre (RHTC) during the study period were screened for eligibility. Participants who met the inclusion criteria were approached by trained investigators and invited to participate in the study. The objectives and procedures of the study were explained in the local language, and written informed consent was obtained from those willing to participate. Recruitment was conducted on all outpatient clinic days throughout the study period, and eligible participants were enrolled consecutively until the target sample size of 550 participants was achieved. No random selection was performed, and every eligible attendee during the recruitment period had an opportunity to participate. Data collection was carried out through interviewer-administered questionnaires and standardized anthropometric measurements.

Anthropometric measurements were collected using standardized procedures. Height and weight were measured to the nearest 0.1 cm and 0.1 kg, respectively, using calibrated equipment, and BMI was calculated as weight (kg)/height² (m²). Waist circumference was measured with a non-stretchable tape at the midpoint between the lower margin of the last palpable rib and the iliac crest.

Assessment of sleep quality and sleep duration

Sleep quality was assessed using the Pittsburgh Sleep Quality Index (PSQI), a validated self-reported instrument consisting of 19 items grouped into seven domains: subjective sleep quality, sleep latency, sleep duration, habitual sleep efficiency, sleep disturbances, use of sleeping medication, and daytime dysfunction. The global PSQI score ranges from 0 to 21, with higher scores indicating poorer sleep quality. Participants with a global PSQI score greater than five were classified as having poor sleep quality, while those with a score of five or less were classified as having good sleep quality [14].

Average nightly sleep duration was obtained from participant responses and categorized into three groups: <6 hours, 6-8 hours, and >8 hours. These categories were chosen based on previous epidemiological studies examining the relationship between sleep duration and metabolic disorders, including fatty liver disease, where 6-8 hours is generally considered the optimal sleep duration for adults, while shorter (<6 hours) and longer (>8 hours) durations have been associated with adverse metabolic outcomes and increased disease risk [17,18].

Assessment of MAFLD risk

Risk of MAFLD was assessed using a validated non-laboratory-based 15-point risk assessment score developed by Lee et al. [15]. The score incorporates age, body mass index, waist circumference, diabetes status, dyslipidemia, physical activity, alcohol consumption among men, and menopausal status among women, with predefined weighted point assignments for each variable. Participants were categorized as having low MAFLD risk (<8 points) or high MAFLD risk (≥8 points) according to the predefined scoring criteria.

Study variables

The primary outcome variable was high MAFLD risk status. The primary exposure variable was sleep quality, categorized as good or poor based on PSQI score. Secondary exposure variables included sleep duration categories. Additional variables collected included age, sex, body mass index, waist circumference, diabetes mellitus, dyslipidemia, physical activity, smoking status, alcohol consumption, dietary pattern, and family history of metabolic disease.

Statistical analysis

Data were entered into Microsoft Excel (Microsoft Corporation, Redmond, WA, USA) and analyzed using IBM SPSS Statistics for Windows, Version 26.0 (Released 2018; IBM Corp., Armonk, NY, USA). No missing data were observed for variables included in the final analysis; therefore, no imputation procedures were required. Continuous variables were expressed as mean ± standard deviation (SD), whereas categorical variables were reported as frequencies and percentages.

Associations between categorical variables were evaluated using the chi-square test. To assess whether sleep quality and sleep duration were independently associated with high MAFLD risk, a multivariable logistic regression model was constructed with high MAFLD risk status as the dependent variable. The model included sleep quality, sleep duration, sex, smoking status, alcohol consumption, and dietary pattern as covariates. Variables constituting the MAFLD risk score (age, body mass index, waist circumference, diabetes mellitus, dyslipidemia, and physical activity) were not included in the regression model to avoid conceptual overlap and mathematical coupling with the outcome measure. Adjusted odds ratios (AORs) with 95% confidence intervals (CIs) were calculated. Statistical significance was defined as a two-tailed p-value less than 0.05.

Ethical considerations

The study protocol was reviewed and approved by the Institutional Ethics Committee of Amrita School of Medicine, Faridabad (Protocol No. AIMS-IEC-BAS-10-25-001). Written informed consent was obtained from all participants before inclusion in the study. Participant confidentiality was safeguarded by assigning unique study identification numbers and removing personal identifiers from the final dataset.

## Results

Participant characteristics

A total of 550 adults were included in the study. The mean age of participants was 42.6 ± 13.5 years, and females constituted 310 (56.4%) of the study population. The mean BMI was 24.8 ± 4.2 kg/m², while the mean waist circumference was 89.4 ± 11.6 cm. Overall, over one-third of the participants (n = 192, 34.9%) had a BMI ≥ 25 kg/m².

Diabetes mellitus and dyslipidemia were present in 78 (14.2%) and 86 (15.6%) participants, respectively, while a quarter (n = 138, 25.1%) reported a family history of metabolic disease. Low physical activity was observed in 214 (38.9%) participants, 71 (12.9%) reported current alcohol consumption, and 64 (11.6%) were current smokers. More than half of the study population followed a non-vegetarian diet (326, 59.3%). The baseline characteristics of the study participants are summarized in Table 1.

**Table 1:** Sociodemographic and clinical characteristics of the study participants (N = 550) Values are presented as mean ± standard deviation (SD) for continuous variables and frequency (percentage) for categorical variables. BMI ≥ 25 kg/m² was considered overweight/obesity according to the study definition.

Characteristic	Value
Age (years), mean ± SD	42.6 ± 13.5
Age group	
18-34 years, n (%)	162 (29.5)
35-49 years, n (%)	208 (37.8)
≥50 years, n (%)	180 (32.7)
Sex	
Male, n (%)	240 (43.6)
Female, n (%)	310 (56.4)
Anthropometric characteristics	
Height (cm), mean ± SD	161.8 ± 8.9
Weight (kg), mean ± SD	64.7 ± 12.6
BMI (kg/m²), mean ± SD	24.8 ± 4.2
Waist circumference (cm), mean ± SD	89.4 ± 11.6
BMI ≥ 25 kg/m², n (%)	192 (34.9)
Clinical characteristics	
Diabetes mellitus, n (%)	78 (14.2)
Dyslipidemia, n (%)	86 (15.6)
Family history of metabolic disease, n (%)	138 (25.1)
Lifestyle characteristics	
Low physical activity, n (%)	214 (38.9)
Current alcohol consumption, n (%)	71 (12.9)
Current smoker, n (%)	64 (11.6)
Non-vegetarian diet, n (%)	326 (59.3)

Sleep characteristics and MAFLD risk profile

Among the 550 participants, 210 (38.2%) were classified as having poor sleep quality (PSQI > 5), whereas 340 (61.8%) demonstrated good sleep quality (PSQI ≤ 5). Regarding sleep duration, the majority of participants reported sleeping 6-8 hours per night (n = 323, 58.7%). A smaller proportion reported sleeping <6 hours (n = 121, 22.0%) or >8 hours per night (n = 106, 19.3%). Thus, approximately 41.3% of participants reported sleep durations outside the recommended 6-8-hour range.

Based on the non-laboratory MAFLD risk assessment score, nearly one-third of the study participants (n = 174, 31.6%) were classified as having a high risk of MAFLD (Table 2).

**Table 2:** Sleep characteristics and MAFLD risk profile among the study participants (N = 550) Sleep quality was assessed using the Pittsburgh Sleep Quality Index (PSQI). Good sleep quality was defined as PSQI ≤ 5, while poor sleep quality was defined as PSQI > 5. Metabolic dysfunction-associated fatty liver disease (MAFLD) risk was estimated using a validated non-laboratory risk assessment score and categorized as low risk (<8 points) or high risk (≥8 points). Values are presented as frequency (percentage).

Variable	n (%)
Sleep quality	
Good sleep quality (PSQI ≤ 5)	340 (61.8)
Poor sleep quality (PSQI > 5)	210 (38.2)
Sleep duration	
<6 hours	121 (22.0)
6-8 hours	323 (58.7)
>8 hours	106 (19.3)
MAFLD risk category	
Low risk (<8 points)	376 (68.4)
High risk (≥8 points)	174 (31.6)

Association between sleep quality and MAFLD risk

A significant association was observed between sleep quality and MAFLD risk. Nearly half the participants with poor sleep quality were classified as having high MAFLD risk (n = 99, 47.1%), whereas only 22.1% (n = 75) with good sleep quality were at a similar risk. Conversely, participants with good sleep quality had a lower MAFLD risk than those with poor sleep quality (77.9% vs 52.9%).

Chi-square analysis showed a statistically significant association between poor sleep quality and high MAFLD risk (χ² = 39.6, p < 0.001) (Table 3).

**Table 3:** Association between sleep quality and MAFLD risk (N = 550) p-values were calculated using the chi-square test (χ² = 39.6). Percentages represent row-wise proportions. Poor sleep quality was defined as PSQI > 5 and good sleep quality as PSQI ≤ 5. MAFLD: metabolic dysfunction-associated fatty liver disease, PSQI: Pittsburgh Sleep Quality Index.

Sleep quality	High MAFLD risk n (%)	Low MAFLD risk n (%)	Total	p-value
Poor sleep quality	99 (47.1)	111 (52.9)	210	<0.001
Good sleep quality	75 (22.1)	265 (77.9)	340	
Total	174 (31.6)	376 (68.4)	550	

Association between sleep duration and MAFLD risk

Sleep duration was significantly associated with MAFLD risk. Participants sleeping <6 hours per night had the highest prevalence of high MAFLD risk (54/121, 44.6%). In comparison, just 92 out of 323 (28.5%) participants sleeping 6-8 hours and 28 out of 106 (26.4%) participants sleeping >8 hours were at high MAFLD risk.

The association between sleep duration and MAFLD risk was statistically significant (χ² = 12.4, p = 0.002). A significant linear trend was observed, indicating increasing MAFLD risk with shorter sleep duration (p-trend < 0.01) (Table 4).

**Table 4:** Association between sleep duration and MAFLD risk (N = 550) p-values were calculated using the chi-square test (χ² = 12.4). Percentages represent row-wise proportions. Sleep duration was categorized as <6 hours, 6-8 hours, and >8 hours per night. MAFLD: metabolic dysfunction-associated fatty liver disease.

Sleep duration	High MAFLD risk n (%)	Low MAFLD risk n (%)	Total	p-value
<6 hours	54 (44.6)	67 (55.4)	121	0.002
6-8 hours	92 (28.5)	231 (71.5)	323	
>8 hours	28 (26.4)	78 (73.6)	106	
Total	174 (31.6)	376 (68.4)	550	

Multivariable logistic regression analysis

As shown in Table 5, both poor sleep quality and short sleep duration remained significantly associated with high MAFLD risk after adjustment for sex, smoking status, alcohol consumption, and dietary pattern. Participants reporting poor sleep quality had 2.68 times higher odds of high MAFLD risk compared with those reporting good sleep quality (AOR: 2.68; 95% CI: 1.82-3.95; p < 0.001). Similarly, participants sleeping <6 hours per night had significantly higher odds of high MAFLD risk than those reporting normal sleep duration (AOR: 1.71; 95% CI: 1.10-2.64; p = 0.017).

Female sex, smoking status, alcohol consumption, and dietary pattern were not significantly associated with high MAFLD risk in the adjusted model. Although smokers, alcohol consumers, and participants following a non-vegetarian diet demonstrated higher odds of high MAFLD risk, these associations did not reach statistical significance. Overall, the findings indicate that poor sleep quality and short sleep duration were independently associated with high MAFLD risk, even after adjustment for selected demographic and lifestyle factors.

**Table 5:** Multivariable logistic regression analysis of sleep-related factors associated with high MAFLD risk among study participants (N = 550) Adjusted odds ratios (AORs) were derived from a multivariable logistic regression model examining the association between sleep-related variables and high MAFLD risk. Variables included in the model were sleep quality, sleep duration, sex, smoking status, alcohol consumption, and dietary pattern. Components of the MAFLD risk score (age, body mass index, waist circumference, diabetes mellitus, dyslipidemia, and physical activity) were excluded from the model to avoid mathematical coupling between predictors and the outcome variable. Statistical significance was defined as p < 0.05. PSQI: Pittsburgh Sleep Quality Index, MAFLD: metabolic dysfunction-associated fatty liver disease.

Variable	Adjusted odds ratio (AOR)	95% CI	p-value
Poor sleep quality (PSQI > 5)	2.68	1.82-3.95	<0.001
Short sleep duration (<6 h)	1.71	1.10-2.64	0.017
Female sex	1.08	0.75-1.56	0.684
Current smoker	1.24	0.74-2.07	0.412
Current alcohol consumption	1.17	0.71-1.94	0.533
Non-vegetarian diet	1.14	0.80-1.61	0.463

Receiver operating characteristic analysis

**Figure 1:**
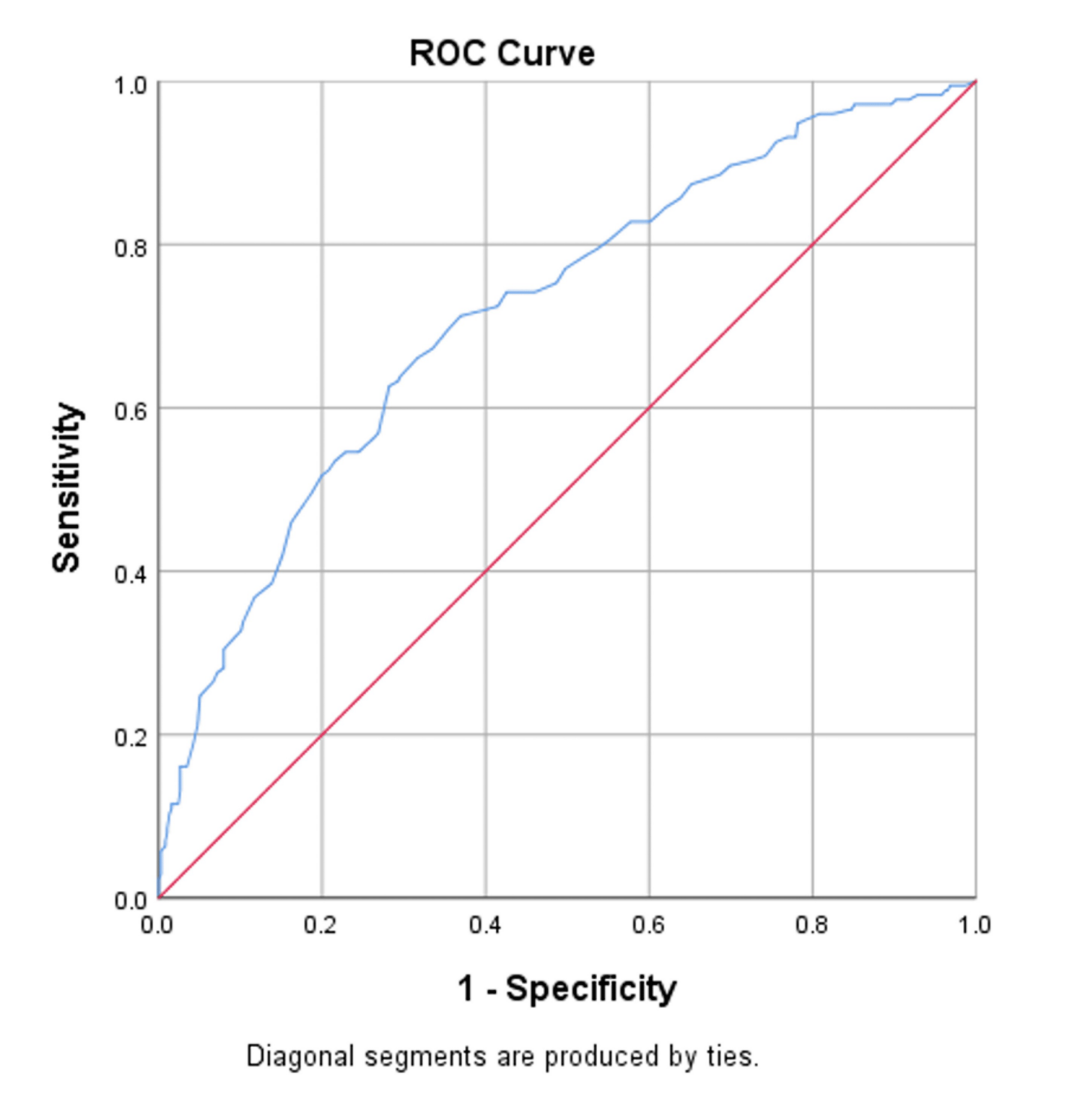
Receiver operating characteristic (ROC) curve of Pittsburgh Sleep Quality Index (PSQI) score for predicting high metabolic dysfunction-associated fatty liver disease (MAFLD) risk The area under the curve (AUC) was 0.71 (95% CI: 0.65-0.77; p < 0.001), indicating acceptable discriminatory ability.

Receiver operating characteristic (ROC) curve analysis was done to evaluate the discriminatory ability of the PSQI score for identifying participants at high risk of MAFLD. The area under the curve was 0.71 (95% CI: 0.65-0.77; p < 0.001), indicating acceptable predictive performance. The ROC curve is shown in Figure 1.

## Discussion

This community-based cross-sectional study found a significant association between sleep-related parameters and high MAFLD risk among adults attending a rural primary healthcare facility in North India. Poor sleep quality was observed in 38.2% of participants, while 31.6% were classified as having high MAFLD risk using a non-laboratory risk assessment score. Participants with poor sleep quality had a substantially higher prevalence of high MAFLD risk than those with good sleep quality. Short sleep duration was also associated with high MAFLD risk, with the highest prevalence observed among individuals sleeping <6 hours per night. On multivariable analysis, both poor sleep quality and short sleep duration remained independently associated with high MAFLD risk after adjustment for selected demographic and lifestyle factors, including sex, smoking status, alcohol consumption, and dietary pattern.

The association between poor sleep quality and elevated MAFLD risk score observed in the present study is consistent with previous epidemiological evidence linking impaired sleep with fatty liver disease. Kim et al. examined sleep duration and sleep quality in relation to MAFLD in a general population and reported that poor sleep quality and short sleep duration were associated with MAFLD, suggesting that sleep-related disturbances may contribute to the metabolic milieu underlying hepatic steatosis [19]. Similarly, Takahashi et al. reported that poor sleep quality was associated with MAFLD, supporting the relevance of subjective sleep assessment in fatty liver disease risk evaluation [20]. Our findings extend these observations to a rural primary care population and suggest that sleep quality may be a useful marker for metabolic liver risk in resource-limited settings.

Short sleep duration was also independently associated with high MAFLD risk in this study. Participants sleeping <6 hours per night had higher odds of being classified as high risk compared with those reporting normal sleep duration. This finding aligns with longitudinal evidence from Okamura et al., who showed that short sleep duration predicted incident MAFLD in a population-based cohort, with an elevated hazard of developing MAFLD among both men and women with shorter sleep duration [11]. Um et al. further demonstrated in a large cohort of more than 140,000 initially MAFLD-free Korean adults that sleep duration and sleep quality were associated with incident MAFLD, reinforcing the potential temporal relationship between sleep disturbance and hepatic steatosis [21]. These findings support the hypothesis that inadequate sleep may be more than a correlate of metabolic disease and may play an independent role in disease development.

The present findings are also consistent with systematic reviews and meta-analyses. A meta-analysis by Wijarnpreecha et al. reported that short sleep duration was associated with an increased risk of MAFLD [22]. More recent evidence by Yang et al. similarly concluded that inadequate sleep duration was strongly correlated with elevated risk of MAFLD/MAFLD [23]. However, the literature remains heterogeneous, with some studies showing stronger associations among women, obese individuals, or metabolically unhealthy subgroups, while others report weaker or non-linear associations. This heterogeneity may be related to differences in study design, population characteristics, ethnicity, methods used to define fatty liver disease, and variation in sleep assessment tools.

Several biological mechanisms have been proposed to explain the observed association between poor sleep and high MAFLD risk. Sleep restriction has been linked to insulin resistance, impaired glucose metabolism, increased sympathetic activity, and dysregulation of appetite-regulating hormones such as leptin and ghrelin [8]. These changes may promote weight gain, central adiposity, and metabolic dysfunction. Sleep disturbances have also been linked to heightened systemic inflammation, characterized by increased levels of inflammatory biomarkers such as interleukin-6 and C-reactive protein, which may promote hepatic injury and disease progression [9]. Circadian rhythm disruption may further impair hepatic lipid metabolism by altering clock gene regulation involved in lipid synthesis, oxidation, and storage. Taken together, these pathways provide biological plausibility for the independent association between sleep disturbances and MAFLD risk.

These findings are relevant from a public health perspective. Rural populations in India are undergoing a rapid metabolic transition, with increasing exposure to sedentary lifestyles, dietary change, obesity, and diabetes mellitus [24]. At the same time, access to ultrasound, transient elastography, and laboratory-based metabolic assessment may be limited in many primary care settings. In this context, simple questionnaire-based tools such as the PSQI, combined with non-laboratory MAFLD risk scores, may help identify individuals who require further evaluation or lifestyle counseling [15]. Sleep assessment may be considered a potentially useful adjunct to routine noncommunicable disease screening, although prospective studies are required to determine its predictive value for future MAFLD risk.

Our study suggests that the PSQI score has an acceptable discriminatory performance for identifying participants at high MAFLD risk. While this does not support the use of PSQI as a standalone diagnostic tool, it indicates that sleep quality may contribute meaningfully to risk stratification. In practical terms, poor sleep quality should be viewed as a potential warning marker that may coexist with other metabolic risk factors and should prompt further assessment of obesity, diabetes, dyslipidemia, physical activity, and liver-related risk.

This study has several strengths. It addresses a relatively underexplored public health question in a rural Indian primary care population. The use of validated tools for both sleep assessment and MAFLD risk estimation makes the approach feasible for community and primary healthcare settings. The study considered several relevant demographic and lifestyle factors, including sex, smoking status, alcohol consumption, and dietary pattern, allowing assessment of whether sleep-related variables remained associated with high MAFLD risk after adjustment for potential confounders.

The study also has its limitations. The cross-sectional design limits causal inference, and the temporal relationship between poor sleep and MAFLD risk cannot be established. Second, MAFLD was not diagnosed using imaging, liver enzymes, or histology. Rather, the study assessed risk using a non-laboratory screening score. Therefore, the findings should be interpreted as associations with high MAFLD risk rather than confirmed MAFLD prevalence. Additionally, because the outcome was derived from a composite MAFLD risk score, variables incorporated into the score were intentionally excluded from the multivariable model to avoid mathematical coupling; therefore, residual confounding by metabolic risk factors cannot be completely excluded. Fourth, unmeasured confounders such as obstructive sleep apnea, shift work, depression, dietary intake, and medication use may have influenced the observed associations. Finally, because the study population was recruited from a rural health training center outpatient department, the findings may have limited generalizability to the wider community.

Despite these limitations, the findings suggest that short sleep duration and poor sleep quality are associated with high MAFLD risk in a rural North Indian primary care population. Incorporating sleep assessment into routine metabolic risk screening may provide a simple and scalable opportunity for early identification of individuals at risk. Further prospective studies using imaging-based or biomarker-confirmed MAFLD outcomes are needed to clarify causality and determine whether improving sleep quality can reduce MAFLD risk or progression.

## Conclusions

Poor sleep quality and short sleep duration were significantly associated with high MAFLD risk among adults attending a rural primary healthcare facility in North India. Participants reporting poor sleep demonstrated a substantially greater likelihood of being classified as high risk for MAFLD, even after adjustment for selected demographic and lifestyle factors. These findings support an association between sleep-related parameters and elevated MAFLD risk and contribute to the growing body of evidence linking sleep and metabolic health.

From a public health perspective, sleep assessment may represent a practical component of comprehensive metabolic health evaluation in primary care settings, particularly in resource-limited environments. Early recognition of sleep disturbances may provide an opportunity for targeted lifestyle interventions before the development of clinically significant liver disease. Further prospective studies incorporating imaging-based or biomarker-confirmed MAFLD outcomes are required to clarify temporal relationships and determine whether sleep-related factors influence future MAFLD risk.
